# Factors Influencing the Use of a Web-Based Application for Supporting the Self-Care of Patients with Type 2 Diabetes: A Longitudinal Study

**DOI:** 10.2196/jmir.1603

**Published:** 2011-09-30

**Authors:** Nicol Nijland, Julia EWC van Gemert-Pijnen, Saskia M Kelders, Bart J Brandenburg, Erwin R Seydel

**Affiliations:** ^1^Department of Psychology, Health and Technology/Center for eHealth Research and Disease ManagementFaculty of Behavioural SciencesUniversity of TwenteEnschedeNetherlands; ^2^MedicinfoTilburgNetherlands

**Keywords:** Internet, technology, eHealth, email, communication, primary care, self-care, diabetes

## Abstract

**Background:**

The take-up of eHealth applications in general is still rather low and user attrition is often high. Only limited information is available about the use of eHealth technologies among specific patient groups.

**Objective:**

The aim of this study was to explore the factors that influence the initial and long-term use of a Web-based application (DiabetesCoach) for supporting the self-care of patients with type 2 diabetes.

**Methods:**

A mixed-methods research design was used for a process analysis of the actual usage of the Web application over a 2-year period and to identify user profiles. Research instruments included log files, interviews, usability tests, and a survey.

**Results:**

The DiabetesCoach was predominantly used for interactive features like online monitoring, personal data, and patient–nurse email contact. It was the continuous, personal feedback that particularly appealed to the patients; they felt more closely monitored by their nurse and encouraged to play a more active role in self-managing their disease. Despite the positive outcomes, usage of the Web application was hindered by low enrollment and nonusage attrition. The main barrier to enrollment had to do with a lack of access to the Internet (146/226, 65%). Although 68% (34/50) of the enrollees were continuous users, of whom 32% (16/50) could be defined as hardcore users (highly active), the remaining 32% (16/50) did not continue using the Web application for the full duration of the study period. Barriers to long-term use were primarily due to poor user-friendliness of the Web application (the absence of “push” factors or reminders) and selection of the “wrong” users; the well-regulated patients were not the ones who could benefit the most from system use because of a ceiling effect. Patients with a greater need for care seemed to be more engaged in long-term use; highly active users were significantly more often medication users than low/inactive users (*P* = .005) and had a longer diabetes duration (*P* = .03).

**Conclusion:**

Innovations in health care will diffuse more rapidly when technology is employed that is simple to use and has applicable components for interactivity. This would foresee the patients’ need for continuous and personalized feedback, in particular for patients with a greater need for care. From this study several factors appear to influence increased use of eHealth technologies: (1) avoiding selective enrollment, (2) making use of participatory design methods, and (3) developing push factors for persistence. Further research should focus on the causal relationship between using the system’s features and actual usage, as such a view would provide important evidence on how specific technology features can engage and captivate users.

## Introduction

The prevalence of diabetes is rising quickly. Diabetes among adults—aged 20–79 years—affected 285 million adults in 2010 (6.4%) and is estimated to increase worldwide to 439 million adults by 2030 (7.7%) [1]. Between 2010 and 2030, the number of adults with diabetes will increase 69% in developing countries and by 20% in developed countries. Most people with diabetes fall within the 60- to 79-year-old age group, and approximately 90% have diabetes mellitus type 2. Improving diabetes care management has therefore become a priority for health care facilities and patients’ organizations worldwide. The ultimate goal of diabetes care management is to optimize self-care in order to reduce mortality, morbidity, and health care costs [2,3].

The introduction of the Internet into clinical practice has brought about many opportunities for self-care [2-7], as it can be used as a powerful medium for promoting a healthy lifestyle and for increasing understanding about the condition. However, to be effective in empowering patients’ self-awareness and engagement, Web applications should be designed to allow individuals to tailor the program to their own specific needs, because patients are increasingly demanding convenient access to a high level of personalized health care [8,9]. To promote self-care, interactive eHealth applications have been developed for continuous self-monitoring, feedback, and information exchange.

From previous studies we know that interactive eHealth technologies contribute positively to health care for patients with a chronic illness, realizing increased patient–provider communication, positive impact on metabolic control and behavior change, improved therapy adherence, and cost reductions [6,7,10-14]. However, the uptake of eHealth in general is still rather low [15,16]. Therefore, more research should be directed toward the factors that provide insights into the actual usage and the accompanying reasons for use and nonuse of eHealth technologies.

Expanding the uptake of eHealth requires, first and foremost, a better understanding of the obstacles that prevent access (initial use) [15-19] and, secondly, a better understanding of the factors that influence the long-term use of eHealth technologies [20-23], since many projects still fail to survive beyond the pilot phase, and user attrition is a typical problem (“Law of Attrition” [22]). To this end, we performed a longitudinal study.

The aim of the study was to explore the factors that influenced the initial and long-term use of a Web-based application for supporting the self-care of patients with type 2 diabetes. A mixed-methods research design was applied to trace the usage over time (log files), along with the reasons for (non)usage (usability tests, interviews, and content analysis of email messages), and to identify user profiles (survey).

## Methods

### Description of the Web Application

DiabetesCoach, a Web-based application for supporting self-care among patients with type 2 diabetes, was developed to encourage patients to play a more active role in their own care. The Web application is a low-tech solution for a large group of patients and was provided free of charge as a supplement to regular diabetes care. The application was developed by Medicinfo (Tilburg, Netherlands) in close collaboration with general practitioners, nurses, patients, behavioral scientists, and vendors (ie, health insurance companies). Initial development costs were relatively limited, and the running costs of the application were low. Therefore, a rise in use would not lead to an exponential rise in costs. 

The following are the core features of the DiabetesCoach:

- *My personal data*: documentation of personal details (eg, treatment plan, medication use).
- *Online monitoring*: registration of metabolic values: weight, blood glucose level, blood pressure, and cholesterol.
- *Email contact*: secured possibility for patient–nurse email communication (response within 5 working days).
- *Online education*: diabetes information and instructions.
- *Calendar*: a place to write down comments, appointments, and personal goals.
- *L* *ifestyle coach*: self-tests to support lifestyle changes.

The patients’ self-monitored data were made available to the nurses with alerts signaling alarming metabolic values. Each nurse had access to each of her own patients’ DiabetesCoach details via a private account (protected by username and password). The Web application (not integrated with the nurse’s medical record) enabled nurses to set individual goals for their patients, add selected lifestyle programs, and highlights the appropriate chapter of the e-learning program. The patients received no particular instructions with regard to how often they should log on to DiabetesCoach. Patients measured metabolic values both at home and at the primary care practice during office visits. Nurses were allowed to have two extra consultation sessions per patient to compensate for the extra time needed to participate in the study. The information and guidelines provided in DiabetesCoach were in accordance with diabetes care standards and protocols in the Netherlands.

### Participant Recruitment

A primary health care foundation in the Netherlands consisting of 10 primary health care practices and a home care organization employing the diabetes nurses (n = 6) agreed to become partners in the pilot. Three primary health care practices volunteered to take part in the DiabetesCoach project.

The selection criteria for patient enrollment were (1) patients with type 2 diabetes mellitus (the primary focus was on fostering lifestyle changes), (2) patients being motivated to perform self-care activities, and (3) patients having access to the Internet and being sufficiently skilled to use the Internet. Through a recruitment letter, 350 patients were invited by the caregivers to use DiabetesCoach. Patients were informed about the purpose and possibilities of the Web application both through the letter and during the office visit. In total, 50 of the 350 invited patients (14%) enrolled in the project and filled out the informed consent forms.

Training sessions (offline) were set up for the enrollees. During the training sessions the participants received instructions on how to use the application, plus a user manual. Also, an email functionality was created for technical support.

### Research Design

**Figure 1:**
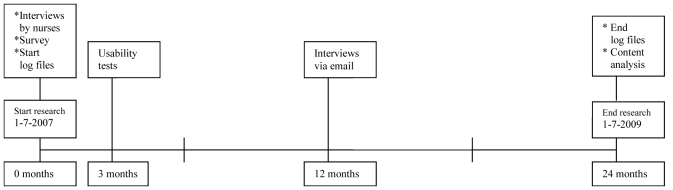
Chronology of the data collection process.

We used a mixed-methods research design [24,25] to explore the conditions for long-term use of a Web application among patients with type 2 diabetes. Through usability tests and interviews we were able to explain the actual usage, and the survey provided insights into who uses the technology. All of the results combined provided an insight into the usage pattern and preferences of individual users for specific technology features. Log files enabled us to assess the actual and long-term usage (24 months) of the technology features. Table 1 presents an overview of the research instruments and the accompanying characteristics of the study. Figure 1 presents a chronology of the data collection process.

Directly after collecting the responses to the invitation letter, the nurses interviewed 226 of the 300 nonenrollees (patients who chose not to participate) during the office visit. Using an open-ended question the nurses asked the nonenrollees about their reasons for nonenrollment.

A *paper-based survey* was administered at baseline during the training sessions of the enrollees (n = 50) to assess patients’ demographics and health-related characteristics (user profiles). The survey consisted of seven closed questions on age, gender, education, health status, diabetes duration, diabetes treatment (medication use), and treatment satisfaction. In total, 42 patients returned the survey completely filled out.

Making use of *log files* (n = 50), we measured the 2-year usage pattern by patients, the number of log-ins by patients, the mean number of hits by patients of the system’s core features, and the content of the patient–nurse email messages.

We performed *u* *sability tests* (n = 20) after 3 months of usage to investigate patients’ experiences with using the Web application. Part 1 of the test consisted of a semistructured interview with open-ended questions aimed at assessing the patients’ eHealth literacy, their reasons for using the Web application, and their positive or negative experiences with using the system based on the critical incidents technique [26,27]. Part 2 contained several tasks related to each feature of the Web application to identify the problems that occurred during real-time use. A trained observer (NN) watched users communicating with the interface of the application while doing simulated tasks and thinking aloud [28]. The participants’ activities were recorded with audiovisual equipment (MORAE version 2.1; TechSmith Corporation, Okemos, MI, USA). The sessions were carried out at the participants’ home or at the health care practice. Each test lasted for about 90 minutes.

One year after the initial use of the Web application, emails were sent to those patients (n = 20) who were not actively using the application by that time. Through an open-ended question patients were asked to report their reason for discontinueing use. We received six responses.

**Table 1:** Research instruments and study characteristics

Research instruments	n	Purpose	Participants
Interviews by nurses	226	Reasons for nonuse of the Web application	Nonenrollees^a^
Survey	50	Who uses the Web application?	Enrollees^b^
Log files	50	What features of the Web application are used?	Enrollees^b^
		Long-term usage pattern (24 months)	
		Profiles of continuous and discontinued users	
Usability tests	20	Reasons for use of the Web application	Enrollees^b^
		Reasons for the decline in usage	
Email interviews	6	Reasons for the decline in usage	Enrollees^b^
Content analysis	50	What sort of information is communicated via the emails?	Enrollees^b^

^a^ Primary care patients who chose *not* to participate in the DiabetesCoach project (n = 300).

^b^ Primary care patients who chose to participate in the DiabetesCoach project (n = 50).

### Data Analysis

#### Statistical Analyses Survey

We performed statistical analyses using SPSS version 16.0 (IBM Corporation, Somers, NY, USA). Standard descriptive statistics were performed, and chi-square tests (Fisher exact test) and *F* tests were used to identify significant differences between the different participant groups—highly active versus low/inactive users—in demographics.

#### Analysis of Interviews by Nurses

The researcher categorized collected responses of patients (n = 226, nonenrollees). Percentages of the answer categories were computed by multiple response analysis.

#### Content Analysis of Email Messages

The coding process of the patient–nurse email messages was based on the grounding theory [29], and the codes of 10 content categories (see Multimedia Appendix 1) that emerged were discussed and classified by two coders (NN, JvG). The unit of analysis contained a single statement reflecting a complete thought or idea [30,31]; this may be expressed as a simple sentence, a sentence clause, a sentence fragment, or a single word. Statements with the same meaning within the same message were coded only once. All email messages were coded independently by NN and JvG. There was 85.7% agreement across categories, with the few instances of disagreement discussed and reconciled. Duplicate messages (n = 12, patient messages) were removed.

#### Analysis of Usability Tests

The usability test data were analyzed using deductive analysis. NN used standard approaches for qualitative data and took detailed notes during the sessions. Notes included the problems experienced during use of the Web application such as poor navigation structures, lack of triggers to use the system, technical errors, and problems with logging on to the system [32,33]. In total, the researcher noted 166 unique problems among 20 patients. Each patient mentioned more than one problem. The coding for problem categories was derived from a conceptual framework developed earlier for the identification of usability problems with eHealth technologies [34].

#### Analysis of User Profiles

To identify the hardcore users we measured the actual use of the Web application by patients (n = 50) during the study period (24 months). Our measure of user activity was defined by three measures: (1) *activity pattern (continuous vs discontinued)*; measures how regularly patients have actually used the Web application until the end of the total study period (Multimedia Appendix 2, Multimedia Appendix 3); (2) *activity degree (high vs low)*; measures for how many months patients have actually used the Web application during the total study period (Multimedia Appendix 3); and (3) *number of log-ins* (Multimedia Appendix 4).

To set the norm for *discontinuity*, we looked at the activity pattern of patients (measure 1). We found that after a period of 7 months of no activity at all, patients began using DiabetesCoach again (see, for example, patient 38 in Multimedia Appendix 2 and Multimedia Appendix 3), but none of the patients did this after 8 months of no activity. In this study we therefore chose to set the norm for discontinuity at 8 months or more of no consecutive activity (Multimedia Appendix 3: search within the activity pattern (nonactive) for the number (8) or higher).

To set the norm for *high activity* (measure 2), we looked for the most active discontinued user (patient 45, Multimedia Appendix 3) and used this users’ activity degree as a cut-off (67%). Continuous users with an activity degree of ≤67% were characterized as *low active users*. Continuous users with an activity degree of >67% were characterized as *highly active (hardcore) users*.

All categories of the user profiles from highly active (7 patients) and low active (10 patients), to inactive users (3 patients) were represented in the usability tests.

## Results

### Nonuse of the Web Application

Only 14% (n = 50) of the 350 patients responded positively to the invitation to use the Web application. Nurses interviewed 226 nonenrollees to gain insight into the barriers that inhibited their enrollment. The reasons given (n = 226) were lack of Internet (146/226, 65%), use will not have any added value (25/226, 11%), not in the mood to spend much time on the computer (23/226, 10%), not in the mood to be occupied with the disease (10/226, 4%), lack of skills to use the Internet (10/226, 4%), too busy or no time (4/226, 2%), or other, such as “patient is about to move to another town” (8/226, 4%). Obviously, patients experienced more external barriers to access (not having the equipment and lacking the right skills: 156/226, 69%) than internal motivational barriers (not willing to use it, no added value, too busy: 62/226, 27%).

### Use of the Web Application

#### Who Uses the Web Application and Why?

The enrollees (n = 50) were aged between 43 and 80 (mean 61) years. Most were male (n = 37), of Dutch origin (40/43, 93%), with a high or medium level of education (Table 2), and treated with a diet and tablets such as metformin. Treatment satisfaction was already high before use of the Web application (40/42, 95%).

Patients mentioned three main reasons for using the Web application:

- *Increased possibilities for self-care*: the system’s features stimulated patients to play a more active role in self-managing their diabetes.
- *More continuously received feedback from the nurse*: patients experienced the feeling of being better looked after by their nurse. The email feature enabled intensified contact between patient and nurse (also in-between the regular trimonthly visits).
- *Improved access to care*: email was convenient for the patients because the nurse was hard to reach by phone.

**Table 2:** Enrollee characteristics

Characteristic			n	%
**Education (n = 43)**				
		Low	5	12
		Medium	22	51
		High	16	37
**Health status (n = 43)**				
		Excellent	0	0
		Very good	6	14
		Good	25	58
		Fair	12	28
		Poor	0	0
**Diabetes treatment (n = 43)**				
		None	2	5
		Diet	4	9
		Diet and tablets	37	86
		Diet, tablets, and insulin	0	0
**Diabetes duration (years) (n = 42)**				
		0–2	12	29
		3–6	16	38
		>7	14	33

#### What Features of the Web Application Are Used?

The log files revealed that the Web application was predominantly used for online monitoring (2216/6289, 35%; total hits of the core features of the Web application by patients during the study period: n = 6289), personal data (1648/6289, 26%) and patient–nurse email contact (1458/6289, 23%), and to a lesser extent for online education (473/6289, 8%), calendar (334/6289, 5%), personal lifestyle coach (160/6289, 3%), and the printing feature (108/6289, 2%). Patients were particularly interested in *online monitoring* for creating measurement overviews (graphs) of their blood sugar levels, weight, and blood pressure (see Multimedia Appendix 5). The *email feature* was used to supplement the online monitoring to provide explanations for their monitored values. The nurse provided weekly feedback to patients and responded to changes in metabolic values and adjusted the treatment regimen (medication) when necessary.

The *personal d* *ata* feature was used together with the online monitoring feature to track medication use to see whether a drug had been effective for improving health. The *c* *alendar* was used to a lesser extent. Instead, email was used to communicate about appointments.

#### What Sort of Information is Communicated Via Emails?

In total, 323 email messages were sent during the study period. In the qualitative content analysis of the email messages, a total of 10 content categories were distinguished (see Table 3 and Multimedia Appendix 1). Certain contrasts were noticed in the content of the patient–nurse email exchange. It turned out that the nurse, more so than the patients, communicated about administrative issues and treatment plans. Communication about treatment plans referred to medication use, with a particular emphasis on medication adjustments. For the nurse the Web application functioned primarily as a means of coordinating care for more efficient communication (time savings, eg, through online appointment scheduling).

Patients, on the other hand, communicated more than nurses about their state of health and how they were feeling. For example, they let their nurse know that they were doing well, as a confirmation or ratification of the treatment regimen. As such, email was primarily used to ensure the nurse was aware of what was going on. Nurses, for their part, responded by giving affective feedback such as expressions of empathy and compliments.

**Table 3:** Email message content by content category quantified by statement^a^

Content categories	Total messages (n = 323)		Patients’ messages (n = 130)		Nurses’ messages (n = 193)	
	n	%	n	%	n	%
Measurements^b^	104	32.2	42	32.3	64	33.2
Administrative communication^c^	101	31.3	25	19.2	77	39.9
Affective communication^d^	99	30.7	38	29.2	63	32.6
DiabetesCoach remarks^d^	49	15.2	28	21.5	21	10.9
Medication use^f^	42	13.0	12	9.2	31	16.1
Physical symptoms^g^	29	9.0	19	14.6	10	5.2
Use of DiabetesCoach functionalities^h^	24	7.4	3	2.3	21	10.9
Lifestyle support^i^	20	6.2	14	10.8	8	4.1
Current events^j^	18	5.6	6	4.6	12	6.2
Other^k^	20	6.2	10	7.7	10	5.2

^a^ Statement = a thematic unit (a unit of meaning within a message); one single message can contain one or more statements.

^b^ Communication about clinical values such as blood sugar, blood pressure, weight, and cholesterol.

^c^ Communication about referrals, appointment scheduling, etc.

^d^ Expression of emotions such as compliments, relief, and worries, as well as social talk (warm wishes and thanks).

^e^ Communication about (technical) problems with the use of the Web application.

^f^ Communication about medication use.

^g^ Communication about physical symptoms/health problems.

^i^ Communication about DiabetesCoach functionalities, other than online monitoring, such as use of the lifestyle coach.

^j^ Communication about new diabetes-related websites and courses.

^k^ Communication not related to the use of the Web application.

### Decline in Usage Over Time

#### Long-Term Usage Pattern

**Figure 2:**
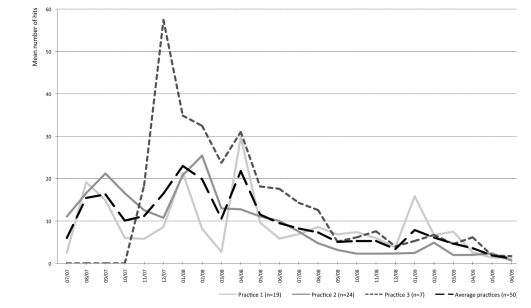
Long-term use of the web application by patients per practice.

Over the total study period (24 months) each patient visited the Web application on average 49 times (2464 hits/50 patients; mean number of log-ins). See Multimedia Appendix 4 for a more detailed insight of the number of log-ins. A decline in usage over time can be observed in all three practices (Figure 2). Practice 3 had a relatively higher overall usage, probably because most technical problems had been solved by the time practice 3 started to use the application (3 months later).

**Figure 3:**
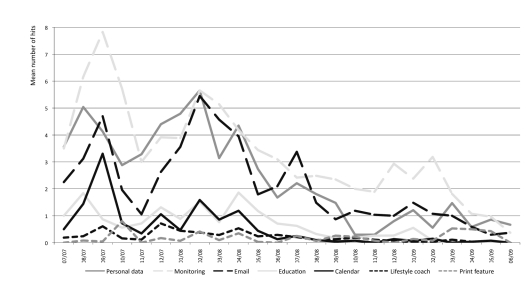
Long-term use of the core features of the web application by patients.

The features personal data, online monitoring, and email contact were all used regularly during the study period (Figure 3).

#### Reasons for the Decline in Usage

Reasons for the decline in usage could be attributed to a *ceiling effect* and *poor user-friendliness* *of the Web application*. The results from the email interviews showed that patients forgot to use the Web application because of the absence of a reminder feature (lack of push factors). The usability tests showed that patients wished to get reminders to use the Web application, preferably through their regular (daily) email program. Patients wished to get notifications on newly posted messages by their nurse and on new and updated information on the site. Integrating the Web application with existing traditional “offline” care could also serve as a push factor. For example, patients with type 2 diabetes can be asked to use the technology for discussing online monitoring during their visit to their general practitioner or nurse.

Patient 17 Perhaps if my diabetes nurse would provide some more help or pay some more attention to it, it might result in more interest.

Patient 1 I wouldn’t mind it being a bit more interactive; that you would get a signal to at least enter something every week and then to get some reply.

The most remarkable observation during the usability test was that the patients were unaware of the possibilities of the system, caused by uncommon navigation structures. In particular, the email feature was undiscovered, which could explain why the message overview was used more extensively than the actual sending of messages (Multimedia Appendix 5). Moreover, the calendar could be more interactive; patients wished to schedule their own appointments via the log book. However, the current log book settings only allowed the nurse to do this.

Furthermore, the email interviews revealed a ceiling effect; for some, using the application no longer had any added value. Patients with their blood sugar level under control had a less pronounced need to use a Web application for self-care support.

Patient 46 Medical checkups have been reduced to twice a year by mutual consultation with my general practitioner. A good result for me personally, but as a result there is very little for me to report.

### Profiles of Highly Active Versus Low/Inactive Users

Multimedia Appendix 2 and Multimedia Appendix 3 present an overview of the monthly use of the Web application during the study period (24 months). It can be seen that use of the Web application fluctuated over time. There was no fixed regimen; each patient used the DiabetesCoach whenever it suited them (free use).

Three groups of users could be distinguished:

(1) *Continuous users who are highly active; hardcore users* (n = 16):

- Activity pattern: period of no activity <8 months (Multimedia Appendix 3)
- Activity degree: 68%–100% (17–24 months use, Multimedia Appendix 3)
- Number of log-ins: 45–191 (Multimedia Appendix 4).

(b) *Continuous users, but with lower levels of activity* (n = 18):

- Activity pattern: period of no activity <8 months
- Activity degree: 29%–67% (7–16 months use)
- Number of log-ins: 10–96.

(c) *Discontinued (inactive) users* (n = 16):

- Activity pattern: period of no activity ≥8 months
- Activity degree: 0%–67% (0–16 months use)
- Number of log-ins: 0–56.

**Figure 4:**
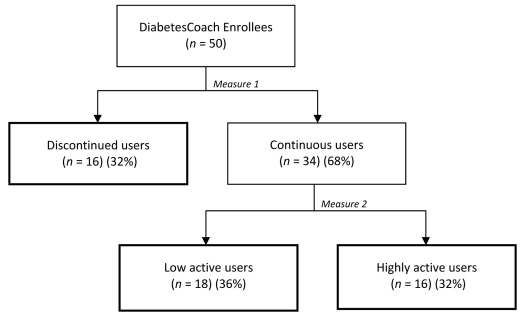
User activity of DiabetesCoach enrollees.

Figure 4 presents user activity over a sustained period of time. About 66% of the enrollees continued using the Web application. Of those regular visitors, 30% can be defined as hardcore users; patients who are highly active in using the Web application.

Multimedia Appendix 2 and Multimedia Appendix 3 show that all patients from practice 1 were continuous users, whereas patients from practice 2 were more likely to be discontinued users. One possible reason for this is the closer contact between the patients and their nurse; the nurse of practice 1 was more actively involved in email contact (interactive feedback) with her patients than the nurses of practice 2 and 3 (respectively 4.5, 3.8, and 2.4 messages sent per patient on average).

When taking into account patient characteristics, the discontinued users did not differ substantially from the continuous users, although more of the discontinued users tended not to be taking medication (11/12, 92%).

We believe that more engagement in system use (being highly active) might result in better adherence to self-care activities. This is why we compared highly active users versus low/inactive users with respect to their characteristics and preferences.

We expected that patients with a greater need for care, such as the elderly, people on medication, and patients who had diabetes for a longer time, would benefit most from the technology and would therefore be more inclined to use the Web application. The results displayed in Table 4 show that highly active users were significantly more often medication users than low/inactive users were (2-sided Fisher exact test, *P* = .005) and had a significantly longer diabetes duration (1-sided analysis of variance, *F*_1__,__41_ = 5.0, *P* = .03).

**Table 4:** Patient characteristics related to user activity

Characteristic			Highly active (n = 16)		Low/inactive (n = 34)		*P* value
			n	%	n	%	
**Gender (n = 50)**							.60
	Male		12	75	25	73	
	Female		4	25	9	26	
**Age (years) (n = 50)**							.28
	43–56		6	37	11	32	
	57–64		7	44	9	26	
	65–80		3	19	14	41	
**Education (n = 43)**							.94
	Low		2	13	3	11	
	Medium		7	47	15	54	
	High		6	40	10	36	
**Health status (n = 43)**							.59
	Very good		3	20	3	11	
	Good		8	53	17	61	
	Fair		4	27	8	29	
**Medication use (n = 43)^a^**							.005
	Yes (tablets)		6	40	1	4	
	No		9	60	27	96	
**Diabetes duration (years) (n = 42)^a^**							.03
	0–2		2	13	10	37	
	3–6		5	33	11	41	
	>7		8	53	6	22	

^a^ *P* < .05

Table 5 presents an overview of the core features and ranks them according to the features that were used most:

- Ranking highly active group: (1) online monitoring, (2) email, (3) personal data.
- Ranking low/inactive group: (1) personal data, (2) online monitoring, (3) email.

Highly active users seemed to have other goals than low/inactive users. Highly active users had a higher need for online monitoring, probably because they were more likely to be frequent medication users who regularly had to pass on their clinical values to their nurse. Particularly for these patients, online monitoring would be convenient (increased access). Low/inactive users, on the other hand, appreciated the ability to document personal details such as treatment plans and medication use (comparable with a personal health record).

The features online monitoring, email, and personal data appealed to both groups, yet the highly active users used all of the features more often, spread over a longer period of time (see Table 6). In particular, they used the interactive features of online monitoring and email more extensively.

**Table 5:** User activity related to the use of system features: ranking of the features

		Personal data^a^	Monitoring	Email	Education	Calendar	Lifestyle coach
**Highly active (n = 16)**							
	Total hits (2 years)	781	1601	908	240	244	96
	Ranking	20%	41%	24%	6%	6%	3%
**Low/inactive (n = 34)**							
	Total hits (2 years)	867	615	550	233	120	64
	Ranking	35%	25%	23%	10%	5%	3%

^a^ Ranking: 20.2% = 781 (total hits personal data)/3870 (total hits of all core features) × 100.

**Table 6:** User activity related to the use of system features: mean number of hits

		Personal data	Monitoring	Email	Education	Calendar	Lifestyle coach
**Highly active (n = 16)**							
	Total hits (2 years)	781	1601	908	240	244	96
	Mean hits per patient^a^	49	100	57	15	15	6
**Low/inactive (n = 34)**							
	Total hits (2 years)	867	615	550	233	120	64
	Mean hits per patient^a^	26	18	16	7	4	2

^a^ Mean hits per patient: 49 = 781 (total hits personal data)/16 (number of highly active patients).

## Discussion

### Main Findings

The aim of this study was to explore the factors that influenced the use of a Web-based application for supporting the self-care of patients with type 2 diabetes. The major advantages of using the Web application were improved access to care and enhanced patient–nurse communication. The features that appealed to the patients most, and with which they were often engaged, were online monitoring in combination with personal feedback through email and documentation of medication usage. These personalized and interactive features stimulated active participation by both the patient and the nurse. Patients felt better monitored by means of the continuously received feedback and were more motivated to take a more active role in self-managing their diabetes.

Unexpectedly, there was a high preference for the documentation of personal data referring to medication and treatment plans. The documentation feature is not interactive; no communication takes place. However, it is comparable, in a certain way, with a personal health record [35-37], as it includes relevant data over the course of the individual’s lifetime. Patients liked to track how medication use affects their health. The personalized aspect means a lot to the patients.

### Factors That Hindered Long-Term Usage of the Web Application

#### Selection Bias

A great concern among eHealth technologies in general, and behavioral intervention programs in particular, is that they may reach those who need them the least (ceiling effect), or they fail to reach the ones with the greatest need for care, such as patients with chronic conditions (inverse care law) [38,39]. Although in the Netherlands the e-patient is taking shape [40], this study’s results still demonstrate a digital divide; the most prominent barrier to enrollment was the lack of Internet access in the patient’s home. Moreover, we found a selective enrollment of relative healthy people: most diabetes patients were well regulated and thus were not the ones who could benefit most from the system. Goldberg et al [5] found similar results in their study; patients felt unengaged because they had already achieved adequate glycemic control. The use of convenience samples should be avoided because it encourages selective enrollment. It attracts patients who are already motivated and who are often the ones who least need the technology.

#### A Ceiling Effect

In the present study a ceiling effect (“I am doing well, so I do not need the technology”) caused attrition. According to Wangberg et al [23], attrition as such is not necessarily a bad thing—in this case it can also be seen as an indicator of success, since the intervention is no longer needed. However, the ceiling effect can have another side to it: because patients do not always have a good insight into their health conditions they might wrongly think that the technology is no longer needed (overestimators). Such a ceiling effect should be avoided. Technology should therefore have persuasive elements such as feedback mechanisms and triggers (eg, email messages) to stimulate users to persist in such cases.

#### Poor User-Friendliness (Lack of “Push” Factors)

The results also illustrate the importance of providing automated reminders, a simple user interface, and personalized content by anticipating the needs of the individual patient. If the patient is not in need of education, then the other features should encourage the patient to use the system. The provision of features with various purposes would be more encouraging to use for a wider audience. Some users asked for the integration of monitoring, recording personal data, and logistics such as scheduling appointments. However, most of the features were presented as stand-alone applications.

### Implications

To foster the widespread use of eHealth technologies like the DiabetesCoach, Internet use should be encouraged among the 65+ age range of the population; it is among the elderly that we have the largest growth potential [41,42]. To do so, the primary health care practices could consider providing training in computer and Internet skills and an opportunity to use a computer with Internet access in the practice itself for those who cannot afford the technology.

Furthermore, we believe that the less motivated or relatively unhealthy patients could benefit the most from the use of eHealth technologies because of their greater need for care and their greater challenge for health improvement. Verheijden et al [39] found that patients with deteriorating health conditions, who are thus more health care dependent, benefitted more from system use and were therefore more inclined to persistently use the Web application. In this study we found evidence, albeit very tentative, for our assumption that use of medication and the duration of the diabetes contributed to technology engagement; highly active users of DiabetesCoach were significantly more often medication users and had significantly longer duration of diabetes. Our results correspond to the findings of Wu et al [43], who found in their study of patients with chronic heart failure that those who used the system had more symptoms. The findings suggest that patients with worse disease conditions are most likely to benefit from eHealth applications. It is therefore expected that the Web application could be most useful for patients who use insulin and have a recent diagnosis of diabetes. Future studies should focus on the encouragement of eHealth among patient populations who can get the most out of it, such as those populations with high rates of behavioral risk factors and multiple chronic conditions [19,44].

In order to understand and overcome technical flaws, users should be able to give feedback during usage so that the system can be fine-tuned to their needs and user profiles. Preferably, users should actively participate in the development of the content (health 2.0) [45]. Patient-centered and -participatory design methods should be used when developing eHealth applications in order to ensure high-quality, user-informed products of demonstrated effectiveness [6,8,46-49]. Through such design approaches we are better able to customize the technology to individual preferences and user profiles. This means that the design of eHealth should start with a careful analysis of individual needs and accompanying system requirements to explore which technology is best suited for whom. 

To increase adherence, technology should have push factors for persistence such as feedback mechanisms and triggers [50]. As such, it is relevant to know what kind of technology features or cues would trigger users, such as through words, images, or sounds. Reminders or triggers for use could be sent via text messages [51] and to the patients’ regular email [52]. Mobile phone technology is gaining ground as a simple interface for the health consumer, given the increasing ubiquity of this technology worldwide, and will therefore be especially useful for patients who seldom use their computer.

Personalized feedback appeared to be one of the most promising features for long-term usage. In fact, two types of personalized feedback via email messages can be distinguished: personalized feedback from a caregiver via secure email and personalized feedback via automated messages and prompts. From the results of this study and the findings of Mohr et al [53] and Fry and Neff [54], we can assume that the use of personalized feedback from a real person is more persuasive than automated tailored feedback. Future research should focus on establishing which type of personalized feedback works best for whom (patients with short-term care needs ie, prevention/cure, versus patients with long-term care needs, ie, chronic disease management) and in which situation (purpose of the communication: task focused versus affective).

Moreover, integrating the technology with existing clinical care could serve as a push factor. Stevens et al [55] found that higher levels of engagement can be reached when technology requires users to log in, for example once a month. Therefore, it is expected that the effects of technology use will be stronger on patients who log in every month (fixed regimen) than on patients who log in only once in a while. By integrating eHealth technology into existing traditional offline care (visits), patients will be triggered to log in within the framework of a fixed regimen.

Besides, education should be provided in a more interactive way, for example through Web 2.0 tools that are built around user-generated or user-manipulated content, such as wikis, blogs, podcasts, and social networking sites [45,56-59].

### Limitations

The limitations of this study include the small and select sample of participants. Users were self-selected, as they were motivated to use the Web application. The patients and nurses who chose to participate in the project may possibly differ from other patient groups. Further research should be conducted, preferably with larger sample groups and among nonenrollees, to gain more thorough insights into the technology preferences of the different patient groups. Nevertheless, we believe that our results provide insights beyond the current literature into patients’ engagement in Web-based disease management programs. The use of a mixed-methods design [24,25] has contributed positively to this. By using interviews and usability tests we were able to explain the actual usage, and the survey provided insights into who used the technology. All of the results combined provided an insight into the usage pattern and preferences of individual users for specific technology features. Log files enabled us to assess the actual and long-term usage of the technology features.

In this study, attrition was not measured with the usual measures, such as Kaplan-Meier [22,60,61]. Most attrition measures analyze survival. However, we could not use these measures in our study because they provide insights only into the drop in usage, and not in the pattern of usage. Such survival curves are perhaps more useful for eHealth interventions with a fixed pattern of use—for example, e-therapy interventions. In our study, the pattern of usage was not fixed. Therefore, we searched for activity patterns in measuring continuity of use and we measured the degree of activity to distinguish between the infrequent users and the highly active users.

### Conclusions

Our findings confirm the need for further research into usage patterns and user profiles [22]. Strategies that engage users with technology are important for addressing the low take-up of eHealth technologies. This study has set out three key strategies for increasing the initial and long-term use of eHealth technologies: (1) avoiding selective enrollment, (2) making use of participatory design methods, and (3) developing push factors for persistence. Innovations in health care will diffuse more rapidly when technology is employed that both is simple to use and has applicable components for interactivity in order to foresee the patients’ need for continuous and personalized feedback, in particular for patients with a greater need for care. More longitudinal research on the use of eHealth technologies such as this study and recently published studies on attrition and adherence factors [52,53,62-70] is needed to provide insights into the way usage fluctuates over time. Through the present study we gained an insight into the differences between highly active users and nonusage dropouts, which can be seen as a first step toward decreasing attrition. The next step could be found when examining the opportunities technology has to offer. Future research should therefore focus on what kinds of system features can increase the use of eHealth technologies.

## Multimedia Appendix 1

Email message content categories.

## Multimedia Appendix 2

Activity pattern of patients (in months).

## Multimedia Appendix 3

User activity (based on activity pattern and activity degree).

## Multimedia Appendix 4

Number of log-ins and number of hits per feature (per patient).

## Multimedia Appendix 5

Number of hits on specific features by patients.
