# Supplementary material for: Factors Influencing the Use of a Web-Based Application for Supporting the Self-Care of Patients with Type 2 Diabetes: A Longitudinal Study
Source: J Med Internet Res. 2011 Sep 30;13(3):e71. doi: 10.2196/jmir.1603 (PMC3222177; doi:10.2196/jmir.1603)
Supplement: Supplementary file 1 [file jmir_v13i3e71_app1.pdf]

Multimedia Appendix. Email message content categories

Code	Category	Example:
1	Affective communication: expression of emotions such as compliments, relief, worries and social talk (wishes, thanks)	Post of patient: At least I am relieved that it has got nothing to do with my diabetes!<br>Post of nurse: I see you've been actively engaged in self-control. It looks really good! Keep it up!
2	Administrative communication: communication about referrals, appointment scheduling, etc.	Post of patient: During the last visit, I received prescriptions for three out of four medicines. That all went well; the medicines were ready and waiting for me at the pharmacy. Now, I only need a prescription for Nedios. I hope to hear from you soon.<br>Post of nurse: We have an appointment on Thursday December 6. Unfortunately, I will not be present at the practice that week. Therefore, I would like to reschedule our appointment to Wednesday, December 12 at 08.15 hrs. Please let me know if this new appointment suits you.
3	Online monitoring: communication about clinical values like blood glucose, blood pressure, weight and cholesterol	Post of patient: Values of the last times:<br>16 Febr.: 6.7; 17 Febr.: 6.3; 19 Febr.: 7.0; 20 Febr.: 7.4<br>After the 8+ of Tuesday, February 18, it has constantly been a bit higher these past few days. Do you think we should do something? Or should I just wait and check my blood sugar level more often?<br>Reply of nurse: Your measured blood sugar values are almost all below 7.0, as we like to see. (…) All in all I don't think we need to do anything.
4	Lifestyle support: communication about nutrition, exercise, etc.	Post of patient: White rice is not recommended, or only occasionally. And yellow rice? And what about fried rice and (Chinese) noodles? Do these products contain a lot of sugar/carbohydrates?<br>Reply of nurse: White rice, but also (Chinese) noodles contain proportionally more carbohydrates, compared with the same weight of potatoes. When I see the dietician, I will ask about yellow rice.
5.	Communication about physical symptoms	Post of patient: Just a question about my feet. I have got a blue toenail now three times in a row after a long-distance run. My running shoes fit perfectly and are not too tight.. Is this a bruise and can it do any harm or is it normal?<br>Reply of nurse: As promised, I forwarded your question to the podotherapist. According to the therapist, it is most probably a bruise caused by pressure on the forefoot. Possibly, the shoes are too short or don't fit the instep, which means the foot can slide in the shoe.
6.	Communication about medication use	Post of nurse: First I have a question for you: are you still on Avandia? Next, given that your blood sugar levels are going down a bit, I would like to propose we increase the insulin. According to my data, you are now injecting 38 EH, the plan is to go to 42 EH. After the increase, please send me a new blood sugar count.<br>Reply of patient: Since I have been injecting, I am not on Avandia anymore. From 26 March onwards, I will inject 42 EH.
7.	Use of DiabetesCoach features such as the lifestyle coach	Post of nurse: Often people eat more - and less healthily - during the (Christmas) holidays. The "healthy nutrition guide" will (again) help you make the right choices.
8.	Current events: communication about new diabetes-related websites and courses	Post of nurse: I want to take this opportunity to ask you the following: The Dutch Institute for Sports and Exercise (NISB) is going to develop an exercise program especially for people with (a high risk of) diabetes.
9.	DiabetesCoach remarks: communication about the (technical) working of the application	Post of patient: During my last check of the web application it turned out that my blood sugar values no longer appeared on the screen. Is it possible the previously entered data are therefore lost?<br>Post of nurse: This is a test. It concerns a new function for simultaneously emailing all DiabetesCoach participants. Please confirm receipt of this message by sending me an email.
10.	Other	Post of nurse: Last week the DiabetesCoach pilot study officially ended. Thanks to your participation much has become clear about the usefulness of this site.

This is a Multimedia Appendix to a full manuscript published in the J Med Internet Res, for full copyright and citation information see http://dx.doi.org/10.2196/jmir.1603
