# Supplementary material for: Factors Influencing the Use of a Web-Based Application for Supporting the Self-Care of Patients with Type 2 Diabetes: A Longitudinal Study
Source: J Med Internet Res. 2011 Sep 30;13(3):e71. doi: 10.2196/jmir.1603 (PMC3222177; doi:10.2196/jmir.1603)
Supplement: Supplementary file 2 [file jmir_v13i3e71_app2.pdf]

Multimedia Appendix. Activity pattern of patients (in months)

a	b	1	2	3	4	5	6	7	8	9	10	11	12	13	14	15	16	17	18	19	20	21	22	23	24
H	1	19	34	11	27	13	10	34	16	9	47	32	29	51	9	10	3	8	4	35	16	12	14	0	0
H	2	8	39	7	1	8	2	8	2	0	45	5	2	2	1	0	20	2	0	2	2	0	0	0	0
H	3	11	50	5	1	20	2	5	15	0	2	2	2	2	16	0	1	2	7	2	5	3	0	0	0
H	4	10	50	9	1	20	5	6	49	3	2	11	44	0	11	0	1	2	12	2	8	0	0	0	0
H	5	81	69	33	1	11	14	0	0	4	7	20	43	1	0	1	2	34	88	5	14	9	0	0	2
H	6	44	43	17	30	10	24	0	5	2	4	2	0	1	1	1	3	0	6	2	0	2	0	0	2
H	11	27	41	3	0	55	8	0	0	4	75	86	52	10	36	29	58	15	26	3	2	13	21	29	0
H	12	13	147	58	34	110	6	0	6	13	4	10	2	0	15	5	12	9	0	19	2	0	21	0	0
H	21	8	36	24	12	35	19	14	20	14	10	24	17	8	20	15	17	13	6	24	15	19	16	21	4
H	24	4	159	257	198	55	47	43	96	37	28	35	23	9	8	32	16	20	24	13	68	0	14	16	6
H	27	61	60	49	9	34	73	70	59	55	34	37	28	42	52	6	17	15	22	13	21	4	0	6	0
H	31	48	27	20	16	31	3	62	61	16	5	3	13	22	3	3	2	0	0	5	0	2	0	0	0
H	44	51	142	104	109	32	43	18	6	56	9	3	15	0	0	6	0	3	3	0	0	8	6	0	0
H	47	34	161	45	9	18	39	27	59	4	15	9	8	7	16	11	7	9	14	0	12	20	0	3	0
H	48	24	22	18	30	32	37	30	10	24	3	0	8	3	7	28	17	6	0	0	3	0	0	0	3
H	49	44	40	12	27	26	16	5	6	13	12	11	9	3	10	3	3	0	13	0	0	11	0	9	3
L	7	33	57	16	6	8	7	0	0	38	0	0	0	1	0	1	2	0	2	2	0	0	0	0	4
L	8	10	27	10	7	7	1	0	0	87	27	0	0	1	6	6	20	2	2	2	0	0	0	0	2
L	9	11	44	6	3	18	5	0	0	3	2	2	0	1	0	1	2	0	2	2	0	0	0	0	2
L	10	12	11	2	2	0	7	0	0	22	2	3	6	0	0	1	2	0	2	2	1	0	0	0	2
L	13	10	14	0	0	3	0	0	0	3	0	1	2	0	2	2	0	0	0	0	2	0	0	0	0
L	14	2	12	0	0	20	0	0	0	3	25	1	2	0	2	13	0	0	0	0	2	0	0	0	0
L	15	27	0	0	2	0	0	0	2	0	1	2	0	2	0	0	0	0	0	2	0	0	0	0	0
L	16	6	4	0	55	20	0	0	3	1	2	2	0	2	2	0	0	0	0	2	0	0	0	0	0
L	17	38	41	8	0	68	2	0	0	1	0	0	2	0	2	13	0	0	0	0	2	0	0	0	0
L	18	7	0	0	1	2	4	0	2	3	2	2	0	71	5	0	0	0	0	17	0	0	0	0	0
L	19	53	3	21	90	5	1	2	3	26	12	42	0	0	0	9	0	0	0	4	1	20	0	0	0
L	20	6	7	0	20	5	0	56	12	0	4	22	9	43	9	2	0	0	0	0	8	9	14	0	0
L	25	1	84	4	1	0	45	15	11	7	0	1	4	0	0	0	0	0	0	5	8	0	12	0	0
L	28	1	0	0	1	0	11	0	0	43	0	3	0	0	4	0	0	0	0	0	0	4	0	0	0
L	32	28	3	3	11	30	3	1	10	2	1	14	3	8	0	0	0	7	0	0	0	0	0	0	0
L	38	9	0	8	4	17	90	43	4	0	7	3	0	0	0	0	0	0	0	5	0	0	6	6	0
L	39	5	0	0	31	19	0	15	105	89	155	104	30	17	0	14	0	0	0	4	0	0	0	0	0
L	40	12	0	0	30	18	11	8	19	19	1	4	0	0	13	0	0	0	4	0	0	0	0	2	0
I	22	6	0	0	3	2	14	12	0	2	2	1	2	0	0	0	0	0	0	0	0	0	0	0	0
I	23	15	8	0	0	3	7	16	0	0	2	2	1	0	0	0	0	0	0	0	0	0	0	0	0
I	26	76	24	10	18	0	16	0	0	0	5	5	0	0	0	0	0	0	0	0	0	0	0	0	0
I	29	6	0	0	0	2	0	0	0	0	0	0	0	0	0	0	0	0	0	0	0	0	0	0	0
I	30	3	0	3	0	0	0	0	0	0	0	0	0	0	0	0	0	0	0	0	0	0	0	0	0
I	33	11	0	7	6	5	0	4	4	0	1	0	0	0	0	0	0	0	0	0	0	0	0	0	0
I	34	30	12	5	0	6	19	29	44	9	1	14	78	15	9	0	3	0	0	0	0	0	0	0	0
I	35	6	4	1	27	0	1	0	0	0	0	0	0	0	0	0	0	0	0	0	0	0	0	0	0
I	36	22	18	3	8	0	0	0	0	0	0	0	0	0	0	0	0	0	0	0	0	0	0	0	0
I	37	20	0	3	31	1	1	3	1	0	21	0	0	0	0	0	0	0	0	0	0	0	0	0	0
I	41	3	0	3	6	0	0	0	0	0	0	0	7	0	0	0	0	0	0	0	0	0	0	0	0
I	42	0	0	0	0	0	0	0	0	0	0	0	0	0	0	0	0	0	0	0	0	0	0	0	0
I	43	24	6	0	19	29	27	74	35	13	6	0	0	0	0	0	0	0	0	0	0	0	0	0	0
I	46	22	3	0	0	0	4	0	0	0	0	3	0	0	0	0	0	0	0	0	0	0	0	0	0
I	45	22	28	33	41	59	40	24	23	24	24	6	9	29	6	3	9	0	0	0	0	0	0	0	0
I	50	38	0	33	5	0	0	3	0	0	0	0	0	0	0	0	0	0	0	0	0	0	0	0	0

a. Column 1: H=highly active (n=16), L=low active (n=18), I=inactive (n=16), b. Column 2: patient number, c. ☐ active ☐ non-active

This is a Multimedia Appendix to a full manuscript published in the J Med Internet Res, for full copyright and citation information see http://dx.doi.org/10.2196/jmir.1603
