# Supplementary material for: Factors Influencing the Use of a Web-Based Application for Supporting the Self-Care of Patients with Type 2 Diabetes: A Longitudinal Study
Source: J Med Internet Res. 2011 Sep 30;13(3):e71. doi: 10.2196/jmir.1603 (PMC3222177; doi:10.2196/jmir.1603)
Supplement: Supplementary file 3 [file jmir_v13i3e71_app3.pdf]

Multimedia Appendix. User activity (based on activity pattern and activity degree)

| Patient | Prac-tice | Activity pattern in months active-(non-active) | Discontinued/ continuous use | Activity degree (number of months active) | User activity |
|---|---|---|---|---|---|
| 1 [a] | 1 | 22-(2) | continuous | 92% (22/24 months) | high |
| 2 [a] | 1 | 8-(1)-5-(1)-2-(1)-2-(4) | continuous | 71% (17/24 months) | high |
| 3 | 1 | 8-(1)-5-(1)-6-(3) | continuous | 79% (19/24 months) | high |
| 4 | 1 | 12-(1)-1-(1)-5-(4) | continuous | 75% (18/24 months) | high |
| 5 | 1 | 6-(2)-5-(1)-7-(2)-1 | continuous | 79% (19/24 months) | high |
| 6 | 1 | 6-(1)-4-(1)-4-(1)-2-(1)-1-(2)-1 | continuous | 75% (18/24 months) | high |
| 7 [a] | 1 | 6-(2)-1-(3)-1-(1)-2-(1)-2-(4)-1 | continuous | 54% (13/24 months) | low |
| 8 [a] | 1 | 6-(2)-2-(2)-7-(4)-1 | continuous | 67% (16/24 months) | low |
| 9 | 1 | 6-(2)-3-(1)-1-(1)-2-(1)-2-(4)-1 | continuous | 63% (15/24 months) | low |
| 10 [a] | 1 | 4-(1)-1-(2)-4-(2)-2-(1)-3-(3)-1 | continuous | 63% (15/24 months) | low |
| 11 [a] | 1 | 3-(1)-2-(2)-15-(1) | continuous | 83% (20/24 months) | high |
| 12 [a] | 1 | 6-(1)-5-(1)-4-(1)-2-(1)-1-(2) | continuous | 75% (18/24 months) | high |
| 13 | 1 | 2-(2)-1-(3)-1-(1)-2-(1)-2-(4)-1-(4) | continuous | 38% (9/24 months) | low |
| 14 | 1 | 2-(2)-1-(3)-4-(1)-2-(4)-1-(4) | continuous | 42% (10/24 months) | low |
| 15 | 1 | 1-(2)-1-(3)-1-(1)-2-(1)-1-(5)-1-(5) | continuous | 29% (7/24 months) | low |
| 16 [a] | 1 | 2-(1)-2-(2)-4-(1)-2-(4)-1-(5) | continuous | 46% (11/24 months) | low |
| 17 [a] | 1 | 3-(1)-2-(2)-1-(2)-1-(1)-2-(4)-1-(4) | continuous | 42% (10/24 months) | low |
| 18 | 1 | 1-(2)-3-(1)-4-(1)-2-(4)-1-(5) | continuous | 46% (11/24 months) | low |
| 19 [a] | 1 | 11-(3)-1-(3)-3-(3) | continuous | 63% (15/24 months) | low |
| 20 [a] | 2 | 2-(1)-2-(1)-2-(1)-6-(4)-3-(2) | continuous | 63% (15/24 months) | low |
| 21 | 2 | 24 | continuous | 100% (24/24 months) | high |
| 22 | 2 | 1-(2)-4-(1)-4-(12) | discontinued | 38% (9/24 months) | inactive |
| 23 | 2 | 2-(2)-3-(2)-3-(12) | discontinued | 33% (8/24 months) | inactive |
| 24 [a] | 2 | 20-(1)-3 | continuous | 96% (23/24 months) | high |
| 25 | 2 | 4-(1)-4-(1)-2-(6)-2-(1)-1-(2) | continuous | 54% (13/24 months) | low |
| 26 | 2 | 4-(1)-1-(3)-2-(13) | discontinued | 29% (7/24 months) | inactive |
| 27 [a] | 2 | 21-(1)-1-(1) | continuous | 92% (22/24 months) | high |
| 28 [a] | 2 | 1-(2)-1-(1)-1-(2)-1-(1)-1-(2)-1-(6)-1-(3) | continuous | 29% (7/24 months) | low |
| 29 | 2 | 1-(3)-1-(19) | discontinued | 8% (2/24 months) | inactive |
| 30 | 2 | 1-(1)-1-(21) | discontinued | 8% (2/24 months) | inactive |
| 31 [a] | 2 | 16-(2)-1-(1)-1-(3) | continuous | 75% (18/24 months) | high |
| 32 | 2 | 13-(3)-1-(7) | continuous | 58% (14/24 months) | low |
| 33 | 2 | 1-(1)-3-(1)-2-(1)-1-(14) | discontinued | 29% (7/24 months) | inactive |
| 34 [a] | 2 | 3-(1)-10-(1)-1-(8) | discontinued | 58% (14/24 months) | inactive |
| 35 [a] | 2 | 4-(1)-1-(18) | discontinued | 21% (5/24 months) | inactive |
| 36 | 2 | 4-(20) | discontinued | 17% (4/24 months) | inactive |
| 37 | 2 | 1-(1)-6-(1)-1-(14) | discontinued | 33% (8/24 months) | inactive |
| 38 [a] | 2 | 1-(1)-6-(1)-2-(7)-1-(2)-2-(1) | continuous | 50% (12/24 months) | low |
| 39 [a] | 2 | 1-(2)-2-(1)-7-(1)-1-(3)-1-(5) | continuous | 50% (12/24 months) | low |
| 40 | 2 | 1-(2)-8-(2)-1-(3)-1-(4)-1-(1) | continuous | 50% (12/24 months) | low |
| 41 | 2 | 1-(1)-2-(7)-1-(12) | discontinued | 17% (4/24 months) | inactive |
| 42 | 2 | (24) | discontinued | 0% (0/24 months) | inactive |
| 43 [a] | 2 | 2-(1)-7-(14) | discontinued | 38% (9/24 months) | inactive |
| 44 | 3 | 12-(2)-1-(1)-2-(2)-2-(2) | continuous | 71% (17/24 months) | high |
| **45** | 3 | 16-(8) | discontinued | 67% (16/24 months) | inactive |
| 46 | 3 | 2-(3)-1-(4)-1-(13) | discontinued | 17% (4/24 months) | inactive |
| 47 | 3 | 18-(1)-2-(1)-1-(1) | continuous | 88% (21/24 months) | high |
| 48 | 3 | 17-(2)-1-(3) | continuous | 75% (18/24 months) | high |
| 49 | 3 | 16-(1)-1-(2)-1-(1)-2 | continuous | 83% (20/24 months) | high |
| 50 | 3 | 1-(1)-2-(2)-1-(17) | discontinued | 17% (4/24 months) | inactive |

[a] Patients who participated in the usability test/interview (n=20, highly active: n=7, low active: n=10, inactive: n=3)

This is a Multimedia Appendix to a full manuscript published in the J Med Internet Res, for full copyright and citation information see http://dx.doi.org/10.2196/jmir.1603
