# Supplementary material for: Factors Influencing the Use of a Web-Based Application for Supporting the Self-Care of Patients with Type 2 Diabetes: A Longitudinal Study
Source: J Med Internet Res. 2011 Sep 30;13(3):e71. doi: 10.2196/jmir.1603 (PMC3222177; doi:10.2196/jmir.1603)
Supplement: Supplementary file 4 [file jmir_v13i3e71_app4.pdf]

Multimedia Appendix 4. Number of log-ins and number of hits per feature (per patient)

Patient	Practice	User activity	Log-ins	Personal data	Monitor-ing	Email	Educa-tion	Calendar	Lifestyle coach
1	1	high	108	63	34	89	17	30	8
2	1	high	45	26	8	14	12	8	3
3	1	high	47	18	29	10	8	3	0
4	1	high	58	63	35	18	19	1	0
5	1	high	106	52	109	49	4	9	1
6	1	high	55	53	26	5	2	0	10
7	1	low	43	39	18	31	8	4	4
8	1	low	50	41	5	42	23	6	9
9	1	low	34	20	5	3	5	1	3
10	1	low	28	6	2	4	4	1	2
11	1	high	147	31	119	94	3	4	0
12	1	high	81	87	16	163	6	25	4
13	1	low	13	1	3	0	6	0	0
14	1	low	19	30	5	6	1	2	4
15	1	low	11	11	2	1	4	0	0
16	1	low	26	22	7	1	13	2	4
17	1	low	25	54	28	11	13	10	4
18	1	low	20	26	10	12	11	2	1
19	1	low	54	78	18	43	11	4	1
20	2	low	50	21	53	42	0	2	7
21	2	high	118	9	186	24	9	6	1
22	2	inactive	17	0	0	0	0	0	0
23	2	inactive	17	4	2	6	0	1	0
24	2	high	191	81	387	200	57	80	9
25	2	low	29	34	33	44	15	12	6
26	2	inactive	28	22	41	0	0	26	1
27	2	high	153	28	104	260	30	12	19
28	2	low	10	11	4	17	4	3	1
29	2	inactive	3	0	0	0	2	0	0
30	2	inactive	2	0	2	0	0	0	0
31	2	high	80	30	87	62	11	6	1
32	2	low	31	24	14	15	3	0	2
33	2	inactive	8	10	5	1	1	1	0
34	2	inactive	56	41	25	69	22	3	0
35	2	inactive	14	7	1	0	0	0	1
36	2	inactive	9	9	16	7	6	1	0
37	2	inactive	11	30	24	2	2	4	1
38	2	low	39	38	24	32	7	8	5
39	2	low	55	171	81	148	52	14	3
40	2	low	25	30	43	2	0	2	1
41	2	inactive	5	1	3	0	5	0	0
42	2	inactive	0	0	0	0	0	0	0
43	2	inactive	51	19	5	97	7	0	1
44	3	high	112	126	161	31	7	42	4
45	3	inactive	96	32	112	28	5	5	3
46	3	inactive	6	9	10	1	0	0	0
47	3	high	127	43	143	25	42	5	22
48	3	high	85	22	95	7	4	2	4
49	3	high	52	49	62	16	9	11	10
50	3	inactive	14	26	14	8	3	6	0
Tot.			2464	1648	2216	1740	473	364	160

a. □ Continuous users, highly active (n=16) □ Continuous users, low active (n=18) □ Discontinued users (n=16)

This is a Multimedia Appendix to a full manuscript published in the J Med Internet Res, for full copyright and citation information see http://dx.doi.org/10.2196/jmir.1603
