# Supplementary material for: Factors Influencing the Use of a Web-Based Application for Supporting the Self-Care of Patients with Type 2 Diabetes: A Longitudinal Study
Source: J Med Internet Res. 2011 Sep 30;13(3):e71. doi: 10.2196/jmir.1603 (PMC3222177; doi:10.2196/jmir.1603)
Supplement: Supplementary file 5 [file jmir_v13i3e71_app5.pdf]

Multimedia Appendix. Number of hits on specific features by patients

Features of the web application	n
Online monitoring (n = 2216)	
• Blood sugar	1109
• Blood pressure	481
• Weight	571
• Cholesterol	109
My personal data (n = 1648)	
• Personal details	335
• Practitioners' details	221
• Medical details	273
• My medication	344
• Annual check-up	241
• Treatment plan – Standards	79
• Treatment plan – Insulin	56
• Treatment plan - Oral medications	99
Email contact (n = 1458)	
• Message overview	1316
• Send message	142
Online education (n = 473)	
• What is diabetes?	64
• High blood pressure	51
• Lifestyle	52
• Treatment	32
• Low blood pressure	40
• Self-care	59
• Daily life	50
• Kidneys	42
• Eyes	16
• Feet	7
• Insulin injection	3
• Blood sugar	22
• Dietician	10
• Hba1c	15
• Smoking cessation	10

This is a Multimedia Appendix to a full manuscript published in the J Med Internet Res, for full copyright and citation information see http://dx.doi.org/10.2196/jmir.1603
